# Development of a Group Organizational Learning Activity Inventory for the Implementation and Sustainability of Evidence-Based Practice in Nursing

**DOI:** 10.1155/2023/8584029

**Published:** 2023-08-21

**Authors:** Keiko Ishii, Yukie Takemura, Naoko Ichikawa

**Affiliations:** ^1^Department of Nursing Administration, Division of Health Sciences and Nursing, Graduate School of Medicine, The University of Tokyo, Tokyo, Japan; ^2^Nursing Department, The University of Tokyo Hospital, Tokyo, Japan

## Abstract

**Purpose:**

This study aimed to develop a group organizational learning activity inventory for evidence-based practice (EBP) institutionalized by hospitals and to verify its reliability and validity.

**Methods:**

A draft inventory was created after verifying content and face validity of draft items developed based on interview data and materials used in EBP implementation. Construct validity and reliability were assessed cross-sectionally through an anonymous self-administered survey conducted via web and printed questionnaires. Construct validity was confirmed using exploratory and confirmatory factor analyses, and internal consistency and convergent/discriminant validity were verified. Inventory structure was determined using the theoretical model based on the hypothetical constructive concept. Temporal stability was assessed two weeks after the initial survey.

**Result:**

A draft inventory comprising 12 factors and 47 items was created based on 95 draft items. Data from 371 nurses across 55 departments in 12 hospitals were analyzed. The inventory comprised 8 factors and 40 items based on the theoretical model. The comparative fit index was 0.86, the Tucker–Lewis index was 0.85, the root mean square error of approximation was 0.09, and standardized root mean square was 0.04. Cronbach's alpha of each factor was ≥0.8, and the temporal stability was moderate.

**Conclusion:**

We developed the group organizational learning activity inventory, comprising 8 factors and 40 items. Though construct validity was low, the reliability and concurrent/convergent validities were confirmed. This inventory contains new factors that measure the group activities to ensure EBP's continuation in hospitals: forming common knowledge, understanding the value of EBP in groups, and fostering ownership thereof. Using this inventory, departments that introduced EBP can evaluate the extent of activities leading to nurses' continuation of EBP and find the improvement point.

## 1. Introduction

Implementation of evidence-based practice (EBP) by nurses can improve patient outcomes [1, 2]; organizational outcomes such as the quality of care, cost-effectiveness, and work environment [3, 4]; and the professional attitudes of nurses and their job satisfaction [5, 6]. Nurses are expected to incorporate EBP into daily practice and ensure its sustainability. Against this background, the introduction and implementation of EBP in nursing routines are encouraged to facilitate nurses' engagement in EBP [7]. However, implementation of EBP for nurses faces challenges. EBP institutionalized by hospitals may be inconsistently implemented, even when introduced as an organizational rule—focus has been limited to its introduction into clinical practice [8]. Many programs introduced by organizations have been dropped after a certain period [9], and no emphasis has been placed on whether EBP is continued after its introduction. Furthermore, nurses find it difficult to continue new practices [10]. This situation hinders the continuous implementation of EBP, making it difficult to fully exploit its benefits. Methods are needed to ensure that nurses continue to implement EBP introduced by hospitals [11]; however, there are few studies on the continuity and sustainability of EBP in nursing [12, 13].

Organizational and managerial factors suggested for EBP continuation include organizational culture, funding, administrative guidance [14, 15], training and infrastructure development [16], changing and adapting practices to local needs [15], educational meetings, and feedback [17]. Activities within the context in which EBPs are implemented also influence the implementation of EBPs [18–20]; in particular, learning in groups is significant [21]. Therefore, the activities of the group and group learning in which EBP is implemented may be effective in the continuous implementation of EBP in nursing. However, existing models and frameworks focus on implementing EBP into daily practice [1, 22, 23] and do not describe specific methods of group learning activities and efforts that should be undertaken by the nursing groups for continuous implementation of EBP. Furthermore, factors suggested to date for EBP continuation lack a group learning perspective [14–16] because no method has been proposed to measure the group's proactive learning in the implementation of EBP and the activity and extent of group learning.

Even if EBP is institutionalized as a rule by the hospital, its continuity may be difficult to implement because the performing group is not regarded as the learning agent, and no consideration is given to the group's activities. Deliberate organizational learning (OL) is essential for effective clinical practice [24]. Therefore, in this study, we regard the group that implements EBP as a learning agent and focus on the nursing group implementing EBP, that is, the group's learning activities in hospital departments/wards. Specifically, we focused on the group's OL during the OL feedback process [25] that could promote the continuation of EBP institutionalized by hospitals. In the feedback process, knowledge institutionalized by an organization is transformed into integrated knowledge within a group, which can be utilized by individuals [25]. This process is known as the knowledge exploitation process [25], and groups' OL represents a part within the feedback process. This group learning integrates information, forming common knowledge and understanding within the group, thus allowing individuals to change their recognition and behavior [25]. In addition, team learning improves learners' attitudes toward tasks [26]. Through group OL activities, that is, ward-level OL activity, nurses might be able to incorporate EBP as group knowledge, and individuals learn within that group, which may promote the nurse's continuous implementation of EBP.

There is a possibility that each department/ward is engaged in a variety of activities with respect to the EBP introduced by the hospital. Therefore, it is possible to quantitatively capture the OL activities of the department/ward for that EBP. However, the OL activities of nurses in departments/wards implementing EBP have not been identified, and no method has been proposed to measure the OL activities of departments/wards in implementing EBP. Thus, an inventory must be developed to quantitatively measure the contents and degree of nursing groups' OL activities related to EBP continuation and evaluate the activities. Such an inventory would make it possible to clarify the activities of hospital departments and wards, continuously implement EBP, and evaluate activity status on a group-by-group basis. By evaluating the content and extent of group OL activities of each department, managers or nurses in charge of the implementation of EBP could plan specific activities for EBP continuation. Thus, we aimed to develop an inventory to evaluate nursing groups' OL activity for the nurses' continuous implementation of EBP and test its validity and reliability.

## 2. Methods

This research was designed as a cross-sectional study consisting of an anonymous self-administered survey using a web questionnaire and a printed questionnaire. The inventory development was performed following the scale development procedure [27].

### 2.1. Development Phase

#### 2.1.1. Identification of the Domains

The OL group process comprises interpretation and integration [25]. Sensemaking and shared understanding are also similar group processes [28, 29]. We searched the Web of Science, PubMed, CINAHL, and Japan Medical Abstracts Society databases using the keywords “organizational learning,” “interpreting,” “integrating,” “interpretation,” “sense-making,” “shared-understanding,” “shared-knowledge,” and “common-understanding” to identify relevant concepts. The search period was from 1960 to June 2020. Consequently, 39 cases were extracted. The concepts that constitute groups' OL were hypothesized as “forming shared understanding/common understanding,” “giving meaning/meaning generation/understanding of meaning,” “improvement/problem-solving,” and “social modeling.” In this study, the groups' OL activities were defined as those activities and efforts leading to EBP continuation, regardless of their intent.

#### 2.1.2. Draft Items

From October 2020 to March 2021, we conducted semi-structured online interviews with thirteen nurses across two hospitals (four nurse managers, eight staff nurses, and one clinical nurse specialist) working in four wards (intensive care unit, community-based integrated care, internal medicine, and recovery). The criterion for ward selection was whether EBP, institutionalized by the hospital, had been implemented within the previous year. Based on the four concepts of group OL, we asked what activities and efforts were undertaken by the wards to ensure EBP continuation (e.g., “Please tell us about an activity or the efforts that have helped all nurses develop a common understanding or deeper understanding of the (^*∗∗∗*^: name of EBPs)”; “Please tell us about the activities and efforts that have helped your department find their own meaning regarding the (^*∗∗∗*^: name of EBPs).” The average interview duration was 57.0 minutes. Furthermore, considering that it is effective to clarify more specific OL activities for groups and wards implementing EBP to effectively understand and evaluate their learning activities, we collected materials (e.g., explanation documents, PowerPoint presentations used in study sessions, etc.) used during and after the introduction of EBP, reviewed their contents, and used them in the qualitative content analysis [30]. After examining the possibility of reinterpretation of coding points between researchers, it was determined that no new category was generated, and theoretical convergence had occurred as a result of item generation. We used MAXQDA2020 for the qualitative analysis. Totally, 12 categories and 94 subcategories were generated. To measure concrete activities, 94 subcategories were used as draft items, and 12 categories were used as factors.

#### 2.1.3. Draft Inventory

To check the content validity of the 94 draft items, an anonymous self-administered questionnaire was completed by three nursing staff and six researchers, who had experience in implementing EBP institutionalized by the hospital either as nursing staff or managers. The item-level content validity index (I-CVI) was calculated [31]; the criterion for item selection was an I-CVI of 0.78 or higher [32]. Seventy-five items met the criteria. The researchers discussed the items' comprehensiveness and examined their selection and integration. However, the number of items was too large with the I-CVI value as a standard. Thus, we (a) adjusted the level of abstraction of the questions by merging similar items (e.g., “No. 43: Prepare EBP-related materials to make it carry during work” and “No. 44: Arrange EBP-related materials in a place that is easy to check during work” were revised to “Keep EBP-related materials in easily accessible locations during working hours”) and (b) removed the items that measured the experiences of individual nurses rather than strictly group activities (e.g., “Department staff and managers understand that there are differences in the acquisition of EBP by individual staff”). A total of 49 items were generated as a draft inventory.

To confirm face validity (whether the draft inventory appears to measure the intended activity and understandability), we conducted online cognitive interviews with five nurses with experience in implementing EBP or who had a master's degree in nursing [33]. Regarding the interview memorandum, the researchers discussed the items that needed to be revised and those that could be integrated. We deleted two similar items: item 13, “The person in charge of EBP in the department explains it to other professionals (e.g., doctors, PTs, caregivers, nursing assistants),” and item 33, “The manager or person in charge of the department confirms the implementation status of EBP in the department.” Finally, a draft inventory comprising 12 factors and 47 items was created (see Table 1).

### 2.2. Reliability and Validity Testing

#### 2.2.1. Sampling and Recruitment

To select EBPs that become a subject for the groups' OL activity, the selection criteria of EBP were as follows: whether EBP was institutionalized by hospitals between April 2020 and April 2021, conducted in the department/ward at the time of the survey, had scientific evidence, had a direct impact on the patient, and was conducted once a day or during the night shift or approximately four times a week. The target EBP was discussed and selected by the researcher and the research person in charge at each hospital. As the EBP being implemented in each ward, 16 EBPs were selected (Table 2).

#### 2.2.2. Participants

This complete survey sampled 620 hospitals with 400 or more beds. All facilities with advanced treatment, regional medical care support, and general hospitals with at least 400 beds were extracted from the medical institution notification information database of each Regional Bureau of Health and Welfare nationwide in Japan (accessed on July 20, 2021), and request letters were sent. Ward selection included those with five or more nurses on staff, excluding the head nurse, and those performing target EBP during the survey. Participants included registered and certified nurses and clinical nurse specialists working in the target ward. During the survey, those on long-term, childcare, or maternity leave were excluded.

#### 2.2.3. Sample Size and Power

A factor analysis of scale development requires 5–10 times the number of samples [34] and a minimum sample size of 200 [35]. As the draft inventory had 47 items, the sample size was estimated as 235, the response rate was 30%, and the required number of distributions was 780. A re-test requires 1–4 times the number of items [36]. For the test-retest reliability, the intra-class correlation coefficient (ICC (2, 1)) was calculated: the ICC of the null hypothesis was 0.5, the ICC of the alternative hypothesis was 0.7, the significance probability was 5%, and the power was set to 80%. The required sample size was 63 [37].

#### 2.2.4. Data Collection

A printed questionnaire or flyer containing a URL and QR code to access the online survey was distributed to each nurse by the nursing manager of the participating wards. For the paper survey, the completed questionnaire was placed in an enclosed envelope, and each respondent returned it directly to the researcher. The first survey lasted two weeks, from October to November 2021 (the paper survey was administered between November 4 and 18, 2021). To verify the temporal stability of the inventory, an online anonymous self-administered questionnaire was conducted after the initial survey with those who consented to participate. Referring to the test-retest interval that had been set in the scale development study of the OL climate [38], we set the interval between initial survey and the test-retest study to two weeks, considering the possibility that OL activities themselves may change if the period of more than one month is allowed.

To ensure the quality of responses in the online survey, consideration was given to duplicate responses, inappropriate responses, and the selection of participants who meet the inclusion criteria. To avoid duplicate responses from the same person, login IDs were issued for the number of participants, and different IDs were listed on each flyer. To exclude inappropriate responses, we also considered the length of the questionnaire to identify responses of less than 3 seconds per item and an expected response time of less than 10 minutes. We also checked respondents who answered the same choices for all items on the development inventory.

#### 2.2.5. Measures

*(1) Group Organizational Learning Activity Draft Inventory*. The draft inventory was rated on a five-point Likert scale (from 1 = scarcely applicable to 5 = highly applicable), with higher scores reflecting a higher perception of activities. In the instructions, we asked about the activities and efforts that the department conducted from the time when “^*∗∗∗*^ (name of EBPs)” was introduced to the department and not necessarily what the individual did. Furthermore, the EBP in the questions was explained to indicate the selected EBP of each department to ensure that participants could answer regardless of whether the selected EBP was recognized. The EBP name was displayed or described in the questionnaire to indicate what was implemented in the department.

*(2) Overall Evaluation of Groups' Activities to Routinize and Continue Evidence-Based Practice*. To verify concurrent validity, we requested an overall evaluation of the group activities to routinize EBP in the department and their activities for nurses' EBP continuation with the following questions: “To what extent does your department carry out activities and efforts to routinize “^*∗∗∗*^ (name of EBPs)”?” and “To what extent does your entire department carry out activities and efforts to enable nurses to continuously implement “^*∗∗∗*^ (name of EBPs)”?” Each of these items was rated on a 10-point scale (from 1 = not implemented at all to 10 = very well implemented). We predicted a moderate or higher positive correlation with each factor of the group OL activity inventory.

*(3) Organizational Learning Subprocess*. Convergent and discriminant validities were tested using the Japanese version of the Organizational Learning Subprocess Measurement Scale [39]. Flores et al. [40] developed the original scale to measure the OL process which consists of five subprocesses: information acquisition, information distribution, information interpretation, information integration, and organizational memory. The reliability and validity of the Japanese version were confirmed among nurses working in Japanese hospitals [39]. Two subscales—information interpretation and information integration—were used to verify convergent validity. As groups' OL activities would promote information interpretation and integration within a group, we expected a moderate positive correlation. Three subscales—information acquisition, information distribution, and organizational memory—were used to verify the discriminant validity. These subscales are part of the OL process; however, they indicate the processes by which the organization obtains new information, how it is transmitted to the group members, and the manner in which the integrated knowledge becomes routine [40, 41]. A weak positive correlation with the group's OL activity was expected.

*(4) Department and Individual Characteristics*. To collect department characteristics, we asked the research staff at the participating hospitals regarding the department type, the number of staff nurses, and when the target EBP was initiated. We collected data on the respondents' age, sex, years of nursing experience, years at the current hospital and current department, number of intra-hospital transfers in the current hospital, and nursing educational background.

#### 2.2.6. Data Analyses

IBM SPSS Statistics version 27 and IBM SPSS AMOS version 27 were used to analyze the data. The significance level was set to *p* < 0.05.

*(1) Item Analysis*. After confirming that the distribution of the 47 items was unimodal, correlation analyses for each item were conducted. The criterion for item reduction was a correlation coefficient between items of 0.7 or more. However, the items with non-overlapping meanings and those necessary for measuring the concept were retained.

*(2) Construct Validity*. Referring to the results of EFA, the inventory structure was determined using the theoretical model based on the hypothetical constructive concept. The Kaiser–Meyer–Olkin measure of sampling adequacy and Bartlett's test of sphericity were used to determine whether the data were appropriate for factor analysis. Then, an exploratory factor analysis (EFA) with unweighted least squares with Promax rotation was conducted by setting the eigenvalue to 1 or more and the factor extraction criteria to the number of hypothetical factors. A confirmatory factor analysis (CFA) with the maximum likelihood method was conducted on the adopted model [42]. The model fit was assessed using the comparative fit index (CFI), Tucker–Lewis index (TLI), root mean square error of approximation (RMSEA), and standardized root mean square residual (SRMR) [43]. To confirm whether the overall or subfactorial score should be calculated, a one-factor model with one latent variable and a higher-order factor model were created, and the CFA was conducted. Akaike's information criterion was used to compare model fit.

*(3) Internal Consistency*. Item-total correlation (I-T correlation) and Cronbach's alpha coefficient (Cronbach's *α*) were calculated. Cronbach's *α* of 0.7 or higher was used as the criterion for internal consistency [44].

*(4) Concurrent, Convergent, and Discriminant Validity*. We examined concurrent, convergent, and discriminant validity using correlation analyses and Spearman's correlation coefficient.

*(5) Temporal Stability*. We calculated the ICC (2, 1) to examine the test-retest reliability [45].

*(6) Verification of the Validity of Group Scores in Indicating Within-Group Agreement*. Aggregation of the scores was required to measure group OL activity as a function of the entire department rather than an aspect of individual perception. The group OL activity score was calculated by dividing the sum of the item scores by the number of items and averaging them for each subscale. The average value of the group was calculated from the score obtained by evaluating the group to which the individual belonged [46] to evaluate the group-level concept. In this study, the ICC (1), ICC (2) [47], and within-group agreement index *r*_wg_ [46] were calculated as the validity criteria for aggregating data obtained from individuals at the group level.

### 2.3. Ethical Considerations

Participants received a letter that stated the purpose, method, risks, and benefits of the study. They were informed that participation was voluntary and that there would be no disadvantages resulting from non-participation. Prior to starting the online questionnaire, they were given the URL and QR code to log in to the online screen, which stated that they could not be identified by their login ID. Moreover, consent was obtained from participants before proceeding. Research materials were distributed to each nurse by the front-line nurse manager of their department. To reduce coercion, the nurse managers were required to explain to the staff nurses that participation was not a work assignment and that the decision or refusal to participate would not affect personnel evaluations. Nurses who participated in the initial and second surveys were given an Amazon gift card (e-mail type) of 300 and 200 yen, respectively. This study was approved by the Research Ethics Commmittee of the Graduate School of Medicine, the University of Tokyo (2020196NI, 2021131NI).

## 3. Results

### 3.1. Participants' Characteristics

**Figure 1:**
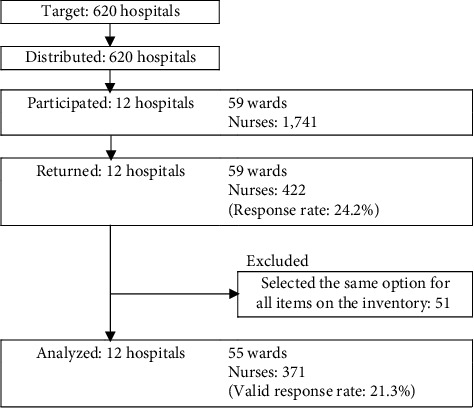
Flowchart of participant selection. The target hospitals were extracted from 620 hospitals that had at least 400 beds and fell under any of the advanced treatment hospitals, regional medical care support hospitals, and general hospitals from the medical institution notification information database of each regional welfare (branch) bureau nationwide.

Of the 620 hospitals that received request letters, 12 consented to participate in the study through the heads of nursing departments. Across 59 wards implementing the EBP at the time of the survey, 1741 nurses were selected, and responses were received from 422 nurses from all 59 departments of the 12 hospitals (response rate of 24.2%). A total of 51 cases that selected the same choices for all items of the inventory were excluded, and the valid responses comprised 371 nurses in 55 departments (Figure 1).  Table 3 lists the hospital and department characteristics.  Table 4 summarizes the participant characteristics.

### 3.2. Item Analysis

Of the 47 items, 7 items (Nos. 7, 17, 19, 35, 39, 41, and 47) were excluded, and 40 were adopted as inventory items. Consequently, social modeling items were deleted from the four hypothetical concepts, and the number of categories was reduced from 12 to 8. The structure of the group OL activity inventory was, thus, assumed to consist of 8 factors and 40 items.

### 3.3. Construct Validity

#### 3.3.1. Exploratory Factor Analysis

Table 5 summarizes the results of the EFA. In the EFA, which set eigenvalues greater than one, the Kaiser standard revealed five factors. The first factor showed an eigenvalue of 24.2, with a cumulative contribution ratio of 59.7%. The scree plot criterion showed a one-factor structure. Various items from three hypothetical concepts converged in the first factor. No items necessary for measuring activity were deleted, even if the factor loading was ≤0.4. In the EFA, which set the factor extraction criteria at eight, the items from various hypothetical concepts were mixed into the first, seventh, and eighth factor. Given that it was difficult to interpret the name of factors, we adopted a theoretical model based on a hypothetical concept.

#### 3.3.2. Confirmatory Factor Analysis

**Figure 2:**
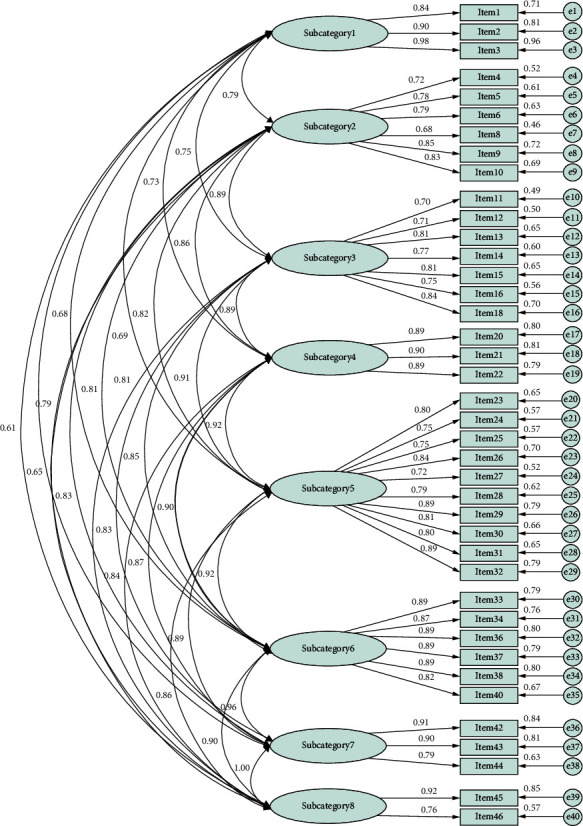
Results of confirmatory factor analysis of the eight-factor model. Model fit statistics: comparative fit index = 0.864, Tucker–Lewis index = 0.852, root mean square of approximation = 0.091 (95% CI: 0.088–0.094), and standardized root mean square residual = 0.044.

The model fit has a CFI of 0.864, TLI of 0.852, RMSEA of 0.091 (95% CI: 0.088–0.094), and SRMR of 0.044 (Figure 2). The covariance between categories was significant.  Table 6 lists the correlation coefficient between factors. The one-factor model and a higher-order factor model were compared, and the eight-factor model showed a better goodness of fit; therefore, the eight-factor structure was adopted for the group OL activities (see Table 7).

### 3.4. Internal Consistency

The I-T correlation ranged from 0.62–0.85. Cronbach's *α* for each of the eight factors was ≥0.8 (Table 6).

### 3.5. Concurrent, Convergent, and Discriminant Validity

Table 8 presents the results of the concurrent, convergent, and discriminant validity analyses. For concurrent validity, the correlations of overall evaluation of groups' activities to routinize and continue EBP and its eight factors were *ρ* = 0.50∼0.59 (*p* < 0.01). For convergent validity, the correlations between information interpretation or integration and its eight factors were *ρ* = 0.19∼0.38 and 0.22∼0.38 (*p* < 0.01). For discriminant validity, correlations between information acquisition and distribution or organizational memory and its eight factors were *ρ* = 0.17∼0.40, 0.22∼0.34, and 0.22∼0.36 (*p* < 0.01).

### 3.6. Temporal Stability

Two weeks after the initial survey, an online questionnaire was conducted on 248 individuals who agreed to participate. In total, 97 responses were received, of which five were excluded because they selected the same choices for all items of the inventory; thus, 92 responses were included in the analysis. The ICC (2, 1) values for each factor were all significant and as follows: 1^st^ factor = 0.63, 2^nd^ factor = 0.54, 3^rd^ factor = 0.52, 4^th^ factor = 0.61, 5^th^ factor = 0.68, 6^th^ factor = 0.65, 7^th^ factor = 0.54, and 8^th^ factor = 0.61.

### 3.7. Verification of the Validity of Group Scores in Indicating Within-Group Agreement

The ICC (1) was 0.18, ICC (2) was 0.73, and *r*_wg_ was 0.59 (range: −0.18–1.00).

## 4. Discussion

Based on the feedback process of OL [25] and the interview, we clarified the contents of the nursing groups' OL activities while implementing the EBP institutionalized by hospitals. We developed an inventory to measure groups' OL activities, consisting of 8 factors and 40 items. Notwithstanding the challenges of EBP continuity, no study has proposed a method for identifying and measuring the activities and efforts undertaken by nursing groups still implementing EBP. This current inventory is a multidimensional and quantitative measure of the group learning activities that enable the sustainability of EBP by stakeholders (departments and wards implementing EBP). This inventory will enable us to visualize the activities of our own department/ward and those of departments/wards that are continuously implementing EBPs. This may enable us to verify which activities are effective. In the following sections, we discuss the quality of the inventory, its limitations, and its strengths.

### 4.1. Concepts and Contents of Groups' Organizational Learning Activity Inventory

Using groups' OL of the feedback process and interview data, we created a draft item inventory based on four hypothetical concepts. However, the “social modeling” items were removed by the item analysis, and the groups' OL activities consisted of eight factors predicated on the following three concepts: “forming shared understanding/common understanding,” “giving meaning/meaning generation/understanding of meaning,” and “improvement/problem-solving.” In terms of the OL theory, social modeling is presented as group OL [48]; however, it might not be implemented as a group's OL activity for EBP continuation.

Although previous studies have clarified the factors necessary for EBP continuation [14–17], they did not measure the autonomous learning activities and lacked the OL perspective. Additionally, the EBP implementation strategy includes reminders, preparation of materials, and consensus-building with the concerned parties [49]. Similar items are contained in this inventory. However, we established new activities to form common knowledge at the group level, understand the significance of EBP, and foster ownership thereof. These could be essential elements for continuing EBP implementation after gaining an understanding of its value and significance, rather than performing it as a mere task. These items are unique to this inventory, which was developed based on OL. Thus, our findings updated content related to specific activities necessary for the continuation of EBP.

### 4.2. Validity and Internal Consistency

Based on the EFA results, the first factor mostly explained the groups' OL activity. Because this is the first inventory to measure groups' OL activity in the feedback process, we prioritized the theoretical factors in CFA to emphasize what kind of factors exist theoretically in the groups' OL activities, and we adopted the eight-factor model based on theoretical structure. The fit of the eight-factor model was low [43]. However, we guaranteed content validity using I-CVI, face validity, and theoretical convergence. As the eight factors of the inventory showed a moderate positive association with the overall evaluation of activities for the routinization and continuation of EBP, concurrent validity was confirmed. In contrast, the information interpretation and integration measured to verify convergent validity had a weaker correlation than expected. Information acquisition, information distribution, and organizational memory were used to verify discriminant validity, showing weak positive correlations. Because the correlation coefficient was small, it was approximately the same as the coefficient of convergent validity. The discriminant validity was not adequately high. A high internal consistency was confirmed in all categories.

### 4.3. Temporal Stability

ICC (2, 1) has moderate reliability when it is between 0.5 and 0.75 [50]; therefore, the temporal stability of this inventory was moderate. The inventory measured the activity of the group; therefore, the degree of activity of each department may have changed depending on the survey time and respondents' evaluations.

### 4.4. Verification of the Validity of Group Scores in Indicating Within-Group Agreement

The cutoff standards for the intra-group consensus index and *r*_wg_ were 0.7 [51]. The *r*_wg_ was low; however, the ICC (1) and ICC (2) showed that the scores of the groups' OL activity had similarities within the group and differences between departments and groups. The average value of the group could also be used to indicate its OL activity.

### 4.5. Research Limitations and Strengths

This study has limitations. First, since the types of EBP at the item generation stage were limited, new categories and items may be generated if another EBP is added in the future. Second, the EBPs in the validity and reliability tests differed from those in the interview; thus, their characteristics might affect the item score and inventory structure. Third, in this study, we employed a large number of items and a theoretical model for our inventory structure as the means to capture the diversity of the group's OL activities. Therefore, reducing the number of items in future research may change the factor structure. Finally, this inventory was developed in the Japanese context, and caution must be exercised if it is to be used in other languages or healthcare systems.

There is a need for effective methods to ensure the continuous implementation of EBP introduced in other medical fields other than nursing. However, few studies have focused on this [12, 13]. Furthermore, in the area of OL research, few empirical studies have been conducted regarding the group-level learning in the feedback process. This has prompted a need to accumulate knowledge on group-level learning [52, 53]. This novel inventory specifically clarifies group OL as an activity within the framework of organizational learning theory, rather than just group learning. As a result, this study contributes to the development of the field of implementation science and organizational learning theory. In addition, the development of implementation strategies is necessary to ensure the sustainable implementation of EBP [54]. The results of the interview showed that several nursing managers and nurses in departments/wards implementing EBP were willing to continue EBP implementation. However, they lacked the knowledge and methods required to ensure EBP's sustainability in their various departments. In this study, the nursing group's implementation of EBP was elaborated as a learning activity and was measurable. This study is innovative because it visualizes the extent of activity and may provide clues for hospital departments/wards to improve their EBP-related efforts and develop EBP implementation strategies.

### 4.6. Recommendations for Further Research

By developing specific activity items from interview data, we developed an inventory to enable strategic management for the continuation of EBP. While this inventory provides a comprehensive picture of the department's learning activities for the continuation of EBPs, the substantial number of items encompassed makes it unsuitable for repeated use. It was not meant for repeated use, such as weekly. Future research should create a shortened version with the eight factors to determine which aspects of the activity are essential even with fewer items, whether reducing the number of factors can still measure the group's organizational learning activities to sustain EBP, and which items are appropriate to measure each factor. Therefore, item reduction in this study was kept to a minimum. In the future, improving the expression of the items, adjusting the number of items, and refining the inventory structure by using various EBPs may be necessary. It is also essential to investigate their relationship with the continuation of EBP that has been introduced in a longitudinal study and verify which factors of the group OL activity inventory are important.

## 5. Conclusions

Based on the feedback process of OL, this study clarified groups' OL activities to sustain EBP. We developed the group OL activity inventory, which consisted of 8 factors and 40 items. This inventory contains the following new factors that can measure the group activities of departments for the continuation of EBP in hospitals by applying OL: forming common knowledge, understanding EBP in groups, and fostering ownership thereof. Although limitations exist, this is the first inventory that can extract a wide range of a group's OL activities and can be used to measure the effectiveness of the group's OL activity for the continuous implementation of EBP. Using our proposed inventory, hospitals that have introduced EBP can evaluate the content and effectiveness of each department's activities and retain effective activities to support EBP continuation.

## Figures and Tables

**Table 1:** List of items in draft inventory.

Hypothetical constructive concept: Forming shared understanding/common awareness	
*First factor: Organizing a team to lead EBP in the unit*	
Item 1	Organize a project team or group that implements EBP-related tasks within the unit
Item 2	Appoint staff members educated in EBP-related tasks as members of the group spearheading EBP in the unit
Item 3	Share aims among members of the project team/group leading EBP in the unit

*Second factor: implementing EBP appropriate for each patient throughout the department/ward*	
Item 4	Hold conferences regularly to evaluate whether the implemented EBP is appropriate
Item 5	Regularly discuss the implemented EBP with other professionals and multidisciplinary teams
Item 6	Request other professionals and multidisciplinary teams to cooperate in the implementation of EBP in the unit
Item 7	Exchange information related to EBPs that are being implemented in patients among nurses and with other professions and professional teams
Item 8	Provide opportunities for unit staff to experience (perform) EBP prior to implementing it for patients
Item 9	Ensure that the EBP project team/group/administrator in the unit checks whether the staff are properly implementing EBP
Item 10	Discuss with the staff whether EBP should be applied to patients

*Third factor: having the same information and knowledge about EBPs among the staff in the department/ward*	
Item 11	Distribute EBP-related materials to each staff member
Item 12	Include EBP-related information in documents perused by all staff members
Item 13	Provide opportunities for staff members to consult with specialists (specialized/certified nurses, professionals with expertise in EBP, medical doctors, etc.) about EBP-related questions
Item 14	Organize multiple EBP briefing sessions
Item 15	The unit manager checks whether each staff member has an acceptable level of awareness when conveying new information to the staff in the unit
Item 16	Use multiple methods (e.g., message notes, e-mail, bulletin board, and mailbox) when communicating new information about EBPs to staff
Item 17	Exchange opinions arising from practicing EBP with other staff members or with experts
Item 18	The EBP project team/group in the unit quantifies and presents the status of EBP implementation in the unit

*Fourth factor: understanding the rationale for EBP and the purpose of implementing it throughout the department/ward*	
Item 19	When explaining the EBP, also explain the department's/ward's challenges and the current status of the department/ward
Item 20	The EBP project team/group/manager in the unit explains the EBP objectives to the staff in simple terms
Item 21	The entire unit reviews whether EBP implementation has been reduced to a formality or whether EBP is being implemented in accordance with the objectives
Item 22	The EBP project team/group/manager in the unit explains to the staff the advantages and disadvantages of EBP implementation

*Fifth factor: implementing EBP consistently among the department/ward staff*	
Item 23	The EBP project team/group in the unit creates EBP-related materials for the staff to carry around during work hours
Item 24	Keep EBP-related materials in easily accessible locations during working hours
Item 25	Include illustrations or photos that help visualize actual usage situations in the EBP materials
Item 26	Formulate EBP rules (conditions, hours for implementation, etc.) that suit the characteristics of the unit
Item 27	Specify when, where, and what to record in the rules within the unit after the EBP is implemented
Item 28	Prepare reference materials for standards and procedures for EBP implementation
Item 29	The person in charge of the unit introduces EBP again a while after its initial introduction
Item 30	Set minimum requirements for EBP implementation that the unit's staff need to meet during the first few months after its implementation
Item 31	The person in charge of the unit repeatedly reminds the staff to implement EBP on multiple occasions (during handover, work hours, end-of-the-day meetings, conferences, etc.)
Item 32	The EBP project team/group clearly explains to the staff what to do before initiating EBP

Hypothetical constructive concept: giving meaning/meaning generation/understanding of meaning	

*Sixth factor: sharing changes by implementing EBP with the entire department/ward*	
Item 33	Share the changes brought about by EBP implementation with the entire unit in an easy-to-understand manner

*Seventh factor: communicating the changes that the EBP will bring to departments/wards and staff*	
Item 34	List the rationale for EBP, challenges in the unit, and expected results in the EBP materials
Item 35	The person or manager in charge of the department communicates to the staff, at an action level, what they seek to achieve beyond the implementation of the EBP
Item 36	The EBP project team/group/manager specifically explains to the staff how EBP will affect patients and what consequences it will have
Item 37	The EBP project team/group/manager in the unit specifically explains to the staff how EBP will change work practices and the benefits it will reap

*Eighth factor: experiencing the effects of EBP with other staff*	
Item 38	Share with the staff the expected results of implementing EBP, as well as the advantages and disadvantages of not implementing EBP
Item 39	Experience with other staff about what EBPs can be used for and what they can lead to

*Ninth factor: experiencing the change across the department/ward for all staff members by implementing EBP*	
Item 40	The manager informs each staff member consecutively regarding the changes brought about by EBP implemented by individual staff members

Hypothetical constructive concept: improvement/problem-solving	

*Tenth factor: supporting staff implementation of EBPs*	
Item 41	Department/ward managers work with staff on factors that make EBP implementation difficult
Item 42	The staff members consult with the administrator about items necessary for EBP implementation
Item 43	The EBP project team/group/manager in the unit proposes the involvement of EBP in the unit's EBP based on the career progression of the staff members
Item 44	The manager stations sufficient staff members to ensure that EBP is implemented every working day

*Eleventh factor*: *adapting EBP to the department/ward*	
Item 45	When reviewing the procedures for EBP, the person in charge of the EBP project team/group listens to the opinions of the staff members who are implementing EBP
Item 46	The nursing department permits the ward to decide about proceeding with EBP at its own discretion

Hypothetical constructive concept: social modeling	

*Twelfth factor: influencing each other mutually as members of the department/ward working with EBPs*	
Item 47	When conducting an EBP, refer to EBPs conducted by other members

**Table 2:** List of selected evidence-based practices (EBPs).

1	Utilization of delirium assessment sheet
2	Utilization of oral health assessment tool (OHAT)
3	Utilization of judgment indicators for serious illness or observation required patient
4	Practices based on a clinical path for delirium preventive care
5	Practices of clean intermittent catheterization (speediCath®)
6	Practices of intensive care unit diaries
7	Nursing intervention based on delirium assessment sheet
8	Implementation of coronavirus infection control measures in line with the nosocomial infection control manual
9	Oral care based on the latest in-hospital standards
10	Pressure ulcer prevention care based on the latest in-hospital standards
11	Infection protection measures based on the latest in-hospital standards
12	Delirium preventive care using CAM-ICU
13	Oral care for patients with endotracheal intubation
14	Intervention based on delirium preventive care guidelines
15	Fixing method of the venous line according to the in-hospital rules
16	Postural change every 4 hours for patients using air mattresses

*Note.* CAM-ICU, Confusion Assessment Method for the Intensive Care Unit. All EBPs were institutionalized by the hospitals.

**Table 3:** Characteristics of hospitals and departments (12 hospitals and 55 departments).

		*n* (%) or mean ± SD
Function	Advanced treatment	2 (16.7)
	Regional medical care support	9 (75.0)
	General	1 (8.3)
Numbers of beds in the hospital		615.7 ± 154.2

Departments	Surgery	12 (21.8)
	Internal medicine	13 (23.6)
	Mixed	23 (41.8)
	Emergency	1 (1.8)
	Operating room	1 (1.8)
	Intensive care unit	4 (7.3)
	Other (emergency visit)	1 (1.8)
Number of nurses in a department		29.9 ± 7.2
Duration from the introduction of the EBP to the department to the survey (in months)		15.3 ± 3.6

**Table 4:** Participant characteristics (*n* = 371).

		*n* (%) or mean ± SD
Age (years)		32.8 ± 9.8

Sex	Male	36 (9.7)
	Female	335 (90.3)

Current occupation	Registered nurse	368 (99.2)
	Associate nurse	1 (0.3)
	Midwife	2 (0.5)

Employment type	Full-time	367 (98.9)
	Non-regular or temporary	2 (0.5)
	Others (contractual part-time)	2 (0.5)

Total years working as a nurse		10.2 ± 9.4
Total years in the current hospital		8.6 ± 8.6
Total years in the current department		3.7 ± 3.9

Nursing education level	Vocational school (associate nurse)	1 (0.3)
	Advanced courses in high school or vocational school	166 (44.7)
	Junior college	28 (7.6)
	Undergraduate	169 (45.6)
	Graduate	7 (1.9)

Experience of intra-hospital transfer	None	186 (50.1)
	Once	79 (21.3)
	More than once	106 (28.6)

**Table 5:** Results of the exploratory factor analysis (*n* = 371).

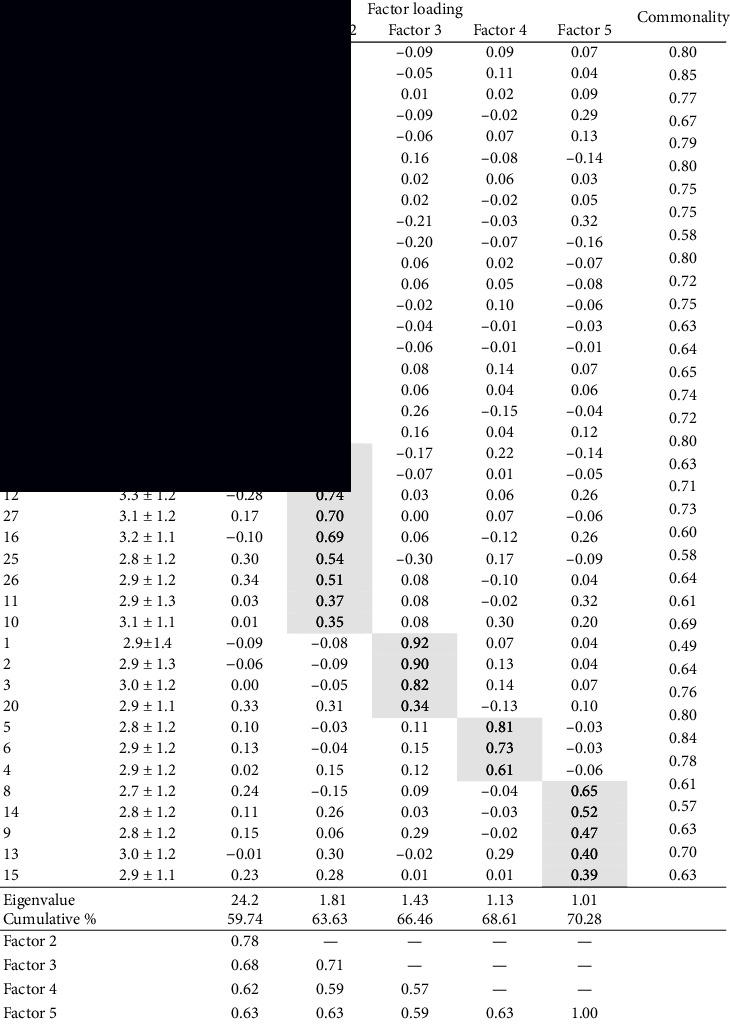

*Note.* Factor extraction method used was unweighted least squares; the criterion for factor extraction was eigenvalue greater than 1; and the rotation method was Promax rotation with Kaiser normalization.

**Table 6:** Results of inter-factor correlations (*n* = 371).

	Factors							
	1	2	3	4	5	6	7	8
Factor 1	(0.93)							
Factor 2	0.80^*∗∗∗*^	(0.90)						
Factor 3	0.75^*∗∗∗*^	0.89^*∗∗∗*^	(0.91)					
Factor 4	0.73^*∗∗∗*^	0.86^*∗∗∗*^	0.89^*∗∗∗*^	(0.92)				
Factor 5	0.71^*∗∗∗*^	0.82^*∗∗∗*^	0.90^*∗∗∗*^	0.92^*∗∗∗*^	(0.95)			
Factor 6	0.69^*∗∗∗*^	0.80^*∗∗∗*^	0.85^*∗∗∗*^	0.88^*∗∗∗*^	0.92^*∗∗∗*^	(0.95)		
Factor 7	0.66^*∗∗∗*^	0.82^*∗∗∗*^	0.83^*∗∗∗*^	0.88^*∗∗∗*^	0.89^*∗∗∗*^	0.95^*∗∗∗*^	(0.90)	
Factor 8	0.64^*∗∗∗*^	0.79^*∗∗∗*^	0.80^*∗∗∗*^	0.85^*∗∗∗*^	0.87^*∗∗∗*^	0.90^*∗∗∗*^	0.97^*∗∗∗*^	(0.82)

*Note.* Spearman's rank correlation, ^*∗∗∗*^*p* < 0.001. The value in the parenthesis refers to Cronbach's alpha.

**Table 7:** Groups' organizational learning activity inventory.

Instruction	These questions ask you to elaborate on the activities or efforts that have been implemented *in your unit to date since the introduction of* [^*∗∗∗*^: name of *EBP*]. These do not necessarily refer to those done by you. Try to recall the activities or efforts that have been *implemented by the unit as a whole* and select the applicable answers. The EBP mentioned in the questions refers to (^*∗∗∗*^: name of EBP) implemented in your unit.
Measurement method	A 5-point Likert scale (1 = scarcely applicable, 2 = not very applicable, 3 = moderately applicable, 4 = fairly applicable, and 5 = highly applicable)
Scoring method	The mean is calculated by category, or through the sum of the mean scores of all categories. Higher scores indicate higher levels of organizational learning activities for that group.

*First factor*	*Organizing a team to lead EBP in the unit*
Item 1	Organize a project team or group that implements EBP-related tasks within the unit
Item 2	Appoint staff members educated in EBP-related tasks as members of the group spearheading EBP in the unit
Item 3	Share aims among members of the project team/group leading EBP in the unit

*Second factor*	*Evaluating the implemented EBP from multiple angles*
Item 4	Hold conferences regularly to evaluate whether the implemented EBP is appropriate
Item 5	Regularly discuss the implemented EBP with other professionals and multidisciplinary teams
Item 6	Request other professionals and multidisciplinary teams to cooperate in the implementation of EBP in the unit
Item 8	Provide opportunities for unit staff to experience (perform) EBP prior to implementing it for patients
Item 9	Ensure that the EBP project team/group/administrator in the unit checks whether the staff are properly implementing EBP
Item 10	Discuss with the staff whether EBP should be applied to patients

*Third factor*	*Ensuring that the staff can acquire common knowledge of EBP*
Item 11	Distribute EBP-related materials to each staff member
Item 12	Include EBP-related information in documents perused by all staff members
Item 13	Provide opportunities for staff members to consult with specialists (specialized/certified nurses, professionals with expertise in EBP, medical doctors, etc.) about EBP-related questions
Item 14	Organize multiple EBP briefing sessions
Item 15	The unit manager checks whether each staff member has an acceptable level of awareness when conveying new information to the staff in the unit
Item 16	Use multiple methods (handover notes, e-mail, bulletin boards, mailboxes, etc.) together when conveying new EBP-related information to the staff
Item 18	The EBP project team/group in the unit quantifies and presents the status of EBP implementation in the unit

*Fourth factor*	*Ensuring that the staff can understand why EBP is being implemented*
Item 20	The EBP project team/group/manager in the unit explains the EBP objectives to the staff in simple terms
Item 21	The entire unit reviews whether EBP implementation has been reduced to a formality or whether EBP is being implemented in accordance with the objectives
Item 22	The EBP project team/group/manager in the unit explains to the staff the advantages and disadvantages of EBP implementation

*Fifth factor*	*Ensuring that the staff can implement EBP in a unified manner*
Item 23	The EBP project team/group in the unit creates EBP-related materials for the staff to carry around during work hours
Item 24	Keep EBP-related materials in easily accessible locations during working hours
Item 25	Include illustrations or photos that help visualize actual usage situations in the EBP materials
Item 26	Formulate EBP rules (conditions, hours for implementation, etc.) that suit the characteristics of the unit
Item 27	Specify when, where, and what to record in the rules within the unit after the EBP is implemented
Item 28	Prepare reference materials for standards and procedures for EBP implementation
Item 29	The person in charge of the unit introduces EBP again a while after its initial introduction
Item 30	Set minimum requirements for EBP implementation that the unit's staff need to meet during the first few months after its implementation
Item 31	The person in charge of the unit repeatedly reminds the staff to implement EBP on multiple occasions (during handover, work hours, end-of-the-day meetings, conferences, etc.)
Item 32	The EBP project team/group clearly explains to the staff what to do before initiating EBP

*Sixth factor*	*Sharing the significance of EBP implementation with the unit and staff*
Item 33	Share the changes brought about by EBP implementation with the entire unit in an easy-to-understand manner
Item 34	List the rationale for EBP, challenges in the unit, and expected results in the EBP materials
Item 36	The EBP project team/group/manager specifically explains to the staff how EBP will affect patients and what consequences it will have
Item 37	The EBP project team/group/manager in the unit specifically explains to the staff how EBP will change work practices and the benefits it will reap
Item 38	Share with the staff the expected results of implementing EBP, as well as the advantages and disadvantages of not implementing EBP
Item 40	The manager informs each staff member consecutively regarding the changes brought about by EBP implemented by individual staff members

*Seventh factor*	*Encouraging the staff to better implement EBP within the unit*
Item 42	The staff members consult with the administrator about items necessary for EBP implementation
Item 43	The EBP project team/group/manager in the unit proposes the involvement of EBP in the unit's EBP based on the career progression of the staff members
Item 44	The manager stations sufficient staff members to ensure that EBP is implemented every working day

*Eighth factor*	*Encouraging the staff to take ownership of EBP*
Item 45	When reviewing the procedures for EBP, the person in charge of the EBP project team/group listens to the opinions of the staff members who are implementing EBP
Item 46	The nursing department permits the ward to decide about proceeding with EBP at its own discretion

**Table 8:** Results of concurrent, convergent, and discriminant validity analyses (*n* = 371).

Groups' organizational learning activity	Overall evaluation of groups' activities	Organizational learning subprocesses				
		Information acquisition	Information distribution	Information interpretation	Information integration	Organizational memory
Factor 1	0.50^*∗∗*^	0.17^*∗∗*^	0.22^*∗∗*^	0.19^*∗∗*^	0.22^*∗∗*^	0.22^*∗∗*^

Factor 2	0.54^*∗∗*^	0.33^*∗∗*^	0.32^*∗∗*^	0.28^*∗∗*^	0.35^*∗∗*^	0.30^*∗∗*^

Factor 3	0.57^*∗∗*^	0.36^*∗∗*^	0.35^*∗∗*^	0.32^*∗∗*^	0.32^*∗∗*^	0.34^*∗∗*^

Factor 4	0.56^*∗∗*^	0.32^*∗∗*^	0.31^*∗∗*^	0.31^*∗∗*^	0.34^*∗∗*^	0.32^*∗∗*^

Factor 5	0.59^*∗∗*^	0.33^*∗∗*^	0.34^*∗∗*^	0.32^*∗∗*^	0.35^*∗∗*^	0.35^*∗∗*^

Factor 6	0.56^*∗∗*^	0.36^*∗∗*^	0.34^*∗∗*^	0.32^*∗∗*^	0.36^*∗∗*^	0.33^*∗∗*^

Factor 7	0.52^*∗∗*^	0.40^*∗∗*^	0.33^*∗∗*^	0.36^*∗∗*^	0.38^*∗∗*^	0.36^*∗∗*^

Factor 8	0.51^*∗∗*^	0.38^*∗∗*^	0.29^*∗∗*^	0.38^*∗∗*^	0.36^*∗∗*^	0.34^*∗∗*^

*Note.* Spearman's rank correlation, ^*∗∗*^*p* < 0.01; factor 1 (organizing a team to lead evidence-based practice (EBP) in the unit), factor 2 (evaluating the implemented EBP from multiple angles), factor 3 (ensuring that the staff can acquire common knowledge of EBP), factor 4 (ensuring that the staff can understand why EBP is being implemented), factor 5 (ensuring that the staff can implement EBP in a unified manner), factor 6 (sharing the significance of EBP implementation with the unit and staff), factor 7 (encouraging the staff to better implement EBP within the unit), and factor 8 (encouraging the staff to take ownership of EBP).

## Data Availability

All data used to support the findings of this study have not been made available because of ethical restrictions.
